# Influences of Sex and Estrogen in Arterial and Valvular Calcification

**DOI:** 10.3389/fendo.2019.00622

**Published:** 2019-09-20

**Authors:** Bin Zhang, Virginia M. Miller, Jordan D. Miller

**Affiliations:** ^1^Department of Surgery, Mayo Clinic, Rochester, MN, United States; ^2^Robert and Arlene Kogod Center on Aging, Mayo Clinic, Rochester, MN, United States; ^3^Department of Physiology and Biomedical Engineering, Mayo Clinic, Rochester, MN, United States; ^4^Department of Cardiovascular Surgery, Mayo Clinic, Rochester, MN, United States

**Keywords:** age-related cardiovascular parameters, aging, aortic valve stenosis, cardiovascular calcification, epidemiology, estrogen, hemodynamics, testosterone

## Abstract

Vascular and cardiac valvular calcification was once considered to be a degenerative and end stage product in aging cardiovascular tissues. Over the past two decades, however, a critical mass of data has shown that cardiovascular calcification can be an active and highly regulated process. While the incidence of calcification in the coronary arteries and cardiac valves is higher in men than in age-matched women, a high index of calcification associates with increased morbidity, and mortality in both sexes. Despite the ubiquitous portending of poor outcomes in both sexes, our understanding of mechanisms of calcification under the dramatically different biological contexts of sex and hormonal milieu remains rudimentary. Understanding how the critical context of these variables inform our understanding of mechanisms of calcification—as well as innovative strategies to target it therapeutically–is essential to advancing the fields of both cardiovascular disease and fundamental mechanisms of aging. This review will explore potential sex and sex-steroid differences in the basic biological pathways associated with vascular and cardiac valvular tissue calcification, and potential strategies of pharmacological therapy to reduce or slow these processes.

## Background

Ectopic calcification in cardiovascular tissue was once considered to be a passive consequence of cardiovascular disease processes with increasing age. The association-based clinical observations driving this model painted a remarkably appealing picture, with vascular calcification being evident in roughly 25% of patients at age of 50 years, and soaring to over 60% in patients over the age of 75 years (1). While the incidence of aortic valve calcification was slightly lower, the overall trend for dramatic, age-associated increases was equally robust. From such population-based studies, risk factors for vascular and valvular calcification quickly emerged, include aging, metabolic syndrome, smoking, and male sex (2–5).

The site-specific mechanisms of ectopic calcification within the tissues of the cardiovascular system are incompletely understood. At present, calcification in either the coronary arteries or cardiac valves is often considered an organized, regulated, and active pathological process, with evidence of many molecular pathways paralleling those observed in bone/orthotopic ossification. Despite this apparent conservation of core osteoblastic signaling pathways, mature bone matrix is rarely found in calcifying cardiovascular tissues (6–8). Critically, however, upstream mechanisms regulating the induction and amplification of these signaling pathways are likely to be fundamentally different between cardiovascular and orthotopic tissues, since exposure to oxidative stress amplifies osteogenic signaling in calcifying cardiovascular cells but markedly suppresses calcification in bone-derived osteoblasts (9). Furthermore, the contribution of dystrophic tissue calcification—where amorphous accumulation of calcium occurs in the absence of bone matrix, functional osteogenic signaling, or presence of osteoblast-like cells—remains remarkably elusive in the pathogenesis of aortic valve stenosis.

In recent years, tissue fibrosis emerged as a potential and major contributor to aortic valve dysfunction in experimental animals. In particular, genetically altered mice with a propensity for both hypertension and hyperlipidemia developed hemodynamically-significant aortic valve stenosis associated with structural and molecular changes consistent with activation of fibrogenic signaling, and critically, in the absence of substantive changes in valvular calcification. Consequently, investigation into the role of fibrosis as a clinically-meaningful determinant of the degree of valvular stenosis is an exciting and emerging field.

## The Clinical Justification for Exploring the Role of Sex in Cavd

While a number of retrospective studies led many to conclude that “women are protected against aortic valve stenosis by estrogens” (10–12), recent work suggests that the pathobiology underlying the disease process may be fundamentally different. For instance, in aortic valve disease—where calcification was once thought to be the primary and near exclusive driver of valve dysfunction–men had more calcification than women at any given level of valvular stenosis (even after normalizing for body size or aortic root size) (13–15), suggesting that valvular fibrosis may play a greater role in determining cusp movement in women compared to men. Similarly, the site of cardiovascular calcification seems to play an important role in predicting mortality in a sex-dependent manner, with thoracic aortic calcification being a strong predictor of mortality secondary to coronary events predominantly in women (16), whereas thoracic aortic and abdominal aortic calcification are strong predictors of all-cause mortality in men (17).

Collectively, the observation that men have higher prevalence of calcification in atherosclerotic lesions and cardiac valves compared to women at any given decade of life has been an interesting clinical observation, and the biological underpinnings and collective clinical implications of these observations are likely to be of great value in developing sex-specific pharmacological treatments to prevent clinically significant valvular and vascular pathology and dysfunction (15, 18, 19). Herein, we aim to highlight potential cellular mechanisms modulated by sex and sex steroid hormones contributing to key sex differences in cardiovascular calcification, with an overall aim of driving dialogue around critical unanswered questions in the field.

## Molecular Signaling involved in Cardiovascular Calcification: aN INITIAL Sex and Sex Hormone Agnostic Perspective

**Figure 1:**
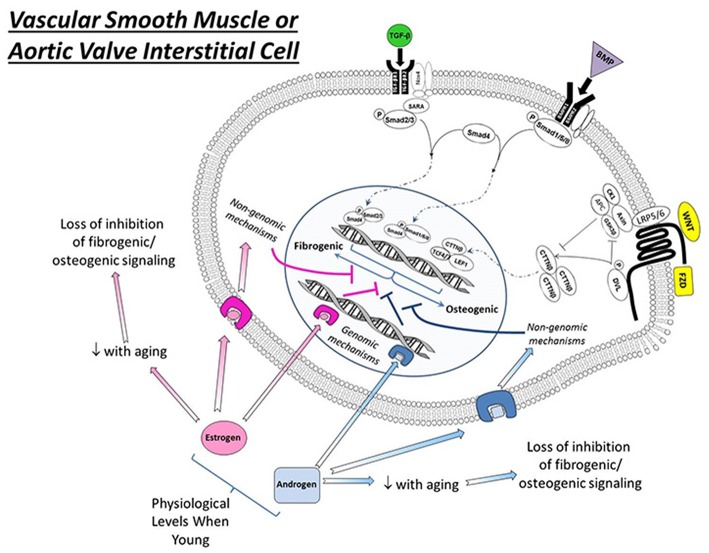
Effects of estrogens and androgen signaling on multiple cellular processes implicated in the regulation of cardiovascular calcification. Note that estrogen can bind to estrogen receptors (ER, resulting in nuclear translation), G-protein coupled estrogen receptors (GPER, eliciting cytosolic signaling), and estrogen binding proteins (EBPs, eliciting cytosolic signaling) to exert a variety of effects—both positive and negative—on molecules influencing ectopic calcification. In general, androgens bind to androgen receptors (AR) and have a smaller number of signal transducing elements compared to estrogens. Abbreviations and impact on calcification: RANKL, receptor activator of nuclear factor κB ligand (promotes calcification); PPAR γ, peroxisome proliferator-activated receptor-γ (prevents calcification); NFκB, nuclear factor κ B (promotes inflammation/calcification); NADPH oxidase, nicotinamide adenine dinucleotide phosphate oxidase (increases oxidative stress/calcification); SOD, superoxide dismutase (reduces oxidative stress/calcification); O_2_•^−^, superoxide anion (increases calcification); p53, tumor protein 53 (promotes inflammation/calcification).

While calcification in the cardiovascular system is often considered an active, regulated process with activation of many fundamental osteogenic signaling cascades being conserved between ectopic cardiovascular calcification and orthotopic bone ossification, upstream mechanisms contributing to the induction, sustained activation, and amplification of these pathways can differ markedly. Interestingly, even during formation of micro-calcific deposits there is expression of proteins which are usually absent (e.g., osteopontin) and/or overexpression of proteins which are usually very low in local tissue (e.g., matrix Gla protein), suggesting that maladaptive processes may be initiated even in the earliest stages of the disease (4, 5, 14, 20, 21). Several of the major molecular signaling pathways involved in regulation of ectopic calcification are ubiquitously active in both males and females (Figure 1).

### Transforming Growth Factor-β (TGF-β) Signaling

One of the most extensively studied pathways in calcific vascular and valvular disease is TGF-β pathway. While the downstream effects are remarkably context dependent (and *in vitro* are dependent on substrate stiffness), TGF-β most often induces cell migration, proliferation, and extracellular matrix protein elaboration. Critically, on stiff substrates and matrices, TGFβ robustly induces apoptosis and dystrophic calcific nodule formation in aortic valve interstitial cells from a variety of species, suggesting that the matrix accumulation and sclerosis occurring in early stages of valvular heart disease may shift the phenotypic consequences of increased TGF-β across the spectrum of the disease and contribute to both fibrosis and dystrophic calcification to different extents during the evolution of disease (22, 23).

### BMP Signaling

Bone morphogenetic proteins (BMPs), members of the TGF-β superfamily, are significantly increased in ectopic calcification lesions within the cardiovascular system including valvular and vascular tissues (22, 24, 25). Initiated by seminal observations from Demer et al., nearly two decades of work have generated compelling data that BMP signaling plays an integral role in the initiation and progression of cardiovascular calcification. Most paradigms implicating BMP signaling suggest that these morphogens serve as a paracrine signal from nearby resident cells to drive osteogenesis via a BMP-Msx2-Wnt cascade (26–28). Importantly, mechanical stimuli—including non-laminar blood flow patterns exacerbated by multiple disease states–induce both oxidative stress and BMP elaboration from vascular and valvular endothelial cells (29, 30). Furthermore, BMP2- and BMP4-driven osteogenic signaling can be further augmented in conditions where endogenous inhibitors (such as matrix Gla protein) are reduced or absent in a variety of disease states (31).

It is noteworthy that not all bone morphogenetic proteins drive ectopic tissue calcification. For example, BMP-7, which is found in human vascular calcification, slows the progression of arterial calcification in both human and mice with diabetes and hyperlipidemia (32, 33). It is also important to note that while the context dependence of TGF-β signaling has been well-defined by numerous investigators, the role of cell-substrate interactions in the phenotypic consequence of BMP signaling has received much less attention in the literature.

### Wnt/β-Catenin Signaling

While increased TGF-β superfamily signaling is a near ubiquitous finding in calcifying cardiovascular tissues, numerous investigators have reported upregulation of other signaling pathways central in bone ossification in diseased vascular and valvular tissues (34). One such pathway is Wnt/β-catenin signaling, where multiple reports have documented increases in Wnt ligand elaboration, low-density lipoprotein receptor-related protein (LRP) receptor components, hyperactivation of canonical β-catenin signaling components, and upregulation of β-catenin transcriptional targets (35, 36).

### The Role of Extracellular Vesicles in Cardiovascular Calcification

Recently, numerous studies have implicated extracellular vesicles in the initiation and progression of cardiovascular calcification (37–39). While their precise role remains largely unclear, several studies reported accumulation of nanoparticles that appear to precede (or occur concomitantly) with induction of osteogenic signals in cardiovascular tissue, and aggregation of such vesicles can contribute to formation of larger calcific masses at multiple cardiovascular sites (40–42). Unlike bone, however, where matrix vesicles are derived largely from chondrocytes and osteoblasts, extracellular vesicles accumulating in the cardiovascular system appear to be derived from vascular smooth muscle cells and/or immune cells/macrophages (43, 44). While the composition of each vesicle subset has yet to be comprehensively characterized, it is likely that the cell origin, mechanism/driver of release, vesicle contents, and target tissue in which deposition occurs are all likely determinants of phenotypic/biological outcomes (45, 46).

### The Site-Specific Role of Inflammation in Ectopic and Orthotopic Calcification

Tumor necrosis factor-α (TNF-α) is a major cytokine involved in driving both local and systemic inflammation in a variety of cardiovascular pathologies. Thus, it is not surprising that upregulation of TNFα has been shown to augment multiple pathophysiological intracellular signaling cascades involved with vascular and valvular calcification, including interactions with BMP signaling, Msx2-dependent gene transcription, and both canonical and non-canonical Wnt/β-catenin signaling) (35, 47–49). For example, the presence of a TNF-α-Msx2–Wnt/β-catenin cascade acts as a major driver of calcium deposition in aortic valve interstitial cells *in vitro* (48, 49) and in atherosclerotic lesions in hyperlipidemic mice *in vivo* (50). While additional mechanistic studies have suggested that antibody-mediated neutralization of TNFα may be effective at slowing initiation or progression of plaque calcification through attenuation of Wnt/β-catenin signaling (3, 50), systemic, long-term suppression of TNFα also puts patients at risk of being immunocompromised, and development of fatal infections.

## Impact of Sex Hormones on Processes of Calcification

Although the overall lifetime incidence of atherosclerosis, aortic stenosis, and cumulative death from cardiovascular diseases are remarkably comparable between men and women (51, 52), the primary contributor to the perception of reduced CVD burden in women is the delay in prevalence of atherosclerosis and/or aortic valve stenosis in women compared to men at each decade of life (53, 54). Our understanding of the impact of sex hormones on cardiovascular calcification is compounded by several factors, including marked differences in sex hormone levels over the lifespan of men and women, the effect, type, and timing of hormone replacement on cardiovascular biology in women, and the general paucity of appropriately powered clinical trial data evaluating differential efficacy and effectiveness of drug interventions on men and women. This understanding is complicated further by the fact that many pre-clinical studies have not actively considered the role of sex in the biological pathways being interrogated (via the exclusion of female animals) or have not faithfully recaptured changes in hormones similar to that observed over the lifespan of humans. Given these caveats, we will address the potential roles of both estrogens and androgens on cardiovascular calcification by first providing limited insights from clinical observations, followed by mechanistic insights gleaned largely from pre-clinical animal models.

## Role of Estrogens in the Regulation of Cardiovascular Calcification

### Clinical Observations

Several seminal studies reported that post-menopausal estrogen treatments may reduce the risk of cardiovascular calcification when administered within the first 5 years of menopause (55, 56). While several recent studies suggest that initiation of estrogen repletion outside of this time period may not confer optimal protection against a myriad of CV complications, the timing, type, and dosing regimens of estrogen that confer vasculo-/valvulo-protection remains a very active field of investigation (57, 58).

### Pre-clnical Observations

Given the increased prevalence of subclinical and clinical CVD occurring within first decade following menopause (59, 60), a large amount of effort has been put into understanding the interplay between exposure to either endogenous or exogenous estrogens and several of the abovementioned pathophysiological signaling cascades.

**Figure 2:**
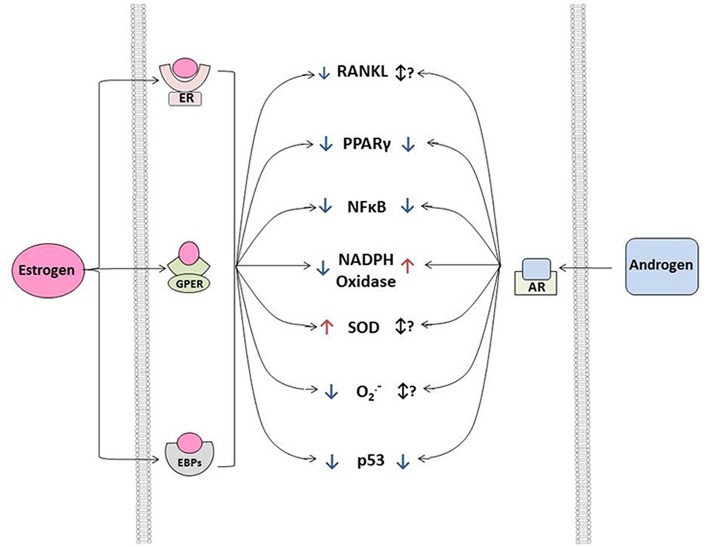
Interactions between estrogen signaling, androgen signaling, and osteogenic signaling in vascular smooth muscle or aortic valve interstitial cells exposed to physiological levels of sex hormones. Note that—in general—both estrogens and androgens suppress osteogenic signaling via both genomic and non-genomic mechanisms in both cell types at physiological levels in relatively early to mid-life stages. Importantly, the therapeutic harnessing of these mechanisms requires substantial research into the context dependence of sex hormone signaling (e.g., timing relative to menopause, level to which hormones should be restored for optimal therapeutic benefit, etc.). TGF-β, transforming growth factor β; BMP, bone morphogenetic protein; Wnt, wingless-related integration site; TGFβR1/2, transforming growth factor beta receptor 1 or 2; BMPR1/2, bone morphogenetic protein receptor 1 or 2; Nox4, NADPH oxidase 4; SARA, smad anchor for receptor activation; Smad, Suppressor of Mothers Against Decapentaplegic; LRP5/6, Low-density lipoprotein receptor-related protein 5 or 6; CK1, Casein kinase 1; DVL, Disheveled protein; Axin, Axin 1 protein; APC, adenomatous polyposis coli protein; GSK3, Glycogen synthase kinase 3; CTTNβ, beta-catenin protein; TCF 4/7, Transcription factor 4 or 7; LEF1, Lymphoid Enhancer Binding Factor 1; FZD, Frizzled receptor.

*in vitro*, estrogen signals through its binding to cytosolic estrogen receptors (such as estrogen receptor α or β), estrogen binding proteins(EBPs), or membrane G-protein-coupled estrogen receptors(Gpr30) (see Figure 2) (61–63). Through a myriad of genomic and non-genomic effects, estrogens can suppress a variety of molecular processes known to drive cardiovascular calcification, including repression of receptor-activator of nuclear factor κB ligand (RANKL) (47, 64) and NFκB signaling, suppression of NADPH oxidase activity in resident cells and inflammatory infiltrates (65, 66) and suppression of p53 (67). Importantly, estrogens do not exert their effects solely by negative regulatory mechanisms, and treatment of cells or animals with exogenous estrogens can drive expression of antioxidant enzymes (in cytosolic, mitochondrial, and lysosomal compartments), and increase nitric oxide synthase activity and expression, both of which have been implicated as key protective mechanisms in cardiovascular calcification (Figure 2) (68–70).

*in vivo*, endogenous estrogen levels are critical for protection against cardiovascular calcification at multiple sites, as surgical ovariectomy in young mice results in accelerated development of advanced calcified lesions in both aortic and aortic valve tissue (14). It is likely that a major mechanism whereby estrogen exerts its protective effects is via the suppression of TGF-β-dependent extracellular matrix production and accumulation and downregulation of non-collagenous proteins (71) in cardiovascular lesions, both of which likely serve to prevent increases in micro-environmental stiffness that increase the propensity for apoptosis in response to sustained elevations in TGF-β (72). The interactions between sex hormones and TGF-β signaling are remarkably complex and context dependent, however, and entire reviews have focused on this topic and unanswered questions in the field (73). Furthermore, given previous work suggesting that TGF-β can reciprocally inhibit estrogen receptor signaling via a canonical smad4 interaction (74, 75), the extent to which systemic estrogens can be increased to win over this interplay remains unclear. Given clinical observations of disproportionately augmented TGF-β signaling and fibrosis in women (which is not associated with increases in deposition of dystrophic calcific deposits) (15), it is evident that the net impact of sex hormones on the molecular and phenotypic sequelae of TGF-β signaling will be paramount to the advancement of pharmacotherapies targeting valvular stenosis.

These estrogenic effects are independent of the Y chromosome, as atherosclerosis, vascular calcification, and bone growth were accelerated in a man with estrogen receptor dysfunction (76). Since testosterone is converted to estrogen by aromatase in both females and males, additional insights related to the role of estrogenic signaling in men can be gleaned from studies in which aromatase inhibition was administered. Here, inhibition of aromatase reduced vascular dilatation in men (77, 78), suggesting the net impact of endogenous “testosterone-derived” estrogens is protective in men. Furthermore, we are not aware of additional clinical evidence that short term use of aromatase inhibitors bring benefit for reducing cardiovascular disease incidence in the elderly male patients with low levels of androgen (i.e., attempts to restore testosterone levels through prevention of its degradation) (78, 79). Complementing these data supporting a net protective effect of aromatase-derived estrogens, administration of aromatase inhibitors for 5 years in women (to reduce the recurrence of estrogen-receptor positive breast cancer), also appears to increase incidence of cardiovascular disease (80).

## Role of Androgens in the Regulation of Cardiovascular Calcification

### Clinical Observations

Numerous studies have shown that, in general, testosterone levels decline relatively linearly after the third decade of life, and can be reduced by more than 50% beyond the sixth decade of life. While the decline in free testosterone is coarsely and inversely related to cardiovascular event rates, causal relationships between changes in testosterone levels and cardiovascular disease remains complicated and highly context dependent (81, 82).

Currently, the vast majority of scientific literature would suggest that supraphysiological levels of testosterone—such as those observed in athletes aiming to improve performance—results in significantly higher levels of coronary artery atherosclerosis compared to non-users of the same age (83–85). Reciprocally, hypogonadal men (testosterone levels <300 ng/dL) have an increased risk of numerous cardiovascular events and complications (86, 87). Of the handful of controlled clinical trials completed to date, most suggest that restoring testosterone levels to mid-normal range does not increase cardiovascular event rates during most follow-up periods (82, 88). Such interventions may, however, increase the volume of non-calcified coronary lesions (89), suggesting that normal levels of testosterone may be pro-atherogenic but not pro-osteogenic/-calcific.

### Pre-clinical Observations

#### Androgens

Numerous studies have probed the interactions between androgen signaling and a variety of pathophysiological signaling cascades in cardiovascular tissues, which have in part been a significant contributor to the controversy surrounding their net impact on cardiovascular diseases. In the context of regulating valvular and vascular disease, several studies have demonstrated a clear role for androgens in the promotion of calcific nodule formation through increasing levels of reactive oxygen species (66, 90–92), repressing PPARγ signaling (93–95), and increasing osteogenic signaling (88, 96, 97) (Figure 2). In line with the aforementioned clinical observations in hypogonadal men, however, physiological levels of androgens may reduce vascular calcification by sustaining eNOS activity (23, 98, 99), reducing TGFβ signaling (100, 101), through the suppression of p53-dependent cellular senescence (67, 102), prevention of cellular apoptosis (91, 103, 104), reducing RANKL signaling (105), suppression of local inflammatory signaling (106–110), and attenuation of pro-thrombotic factor activity (111, 112) (Figure 2).

## Protective Androgens vs. Protective Androgen-derived Estrogens

Similar to aging humans, lower serum testosterone is associated with increased risk of cardiovascular calcification in experimental animals and is attenuated by long-term androgen repletion (98, 113). The biological interpretation of this effect is complicated, however, given the fact that both endogenous and exogenous testosterone can be converted to estrogen by aromatase enzymes (114, 115). Seminal studies showing that androgen receptor-dependent signaling has a deleterious impact on CV calcification (via genetic inactivation of the androgen receptor) combined with reports of augmented vascular calcification in men with spontaneous loss-of-function mutations in estrogen receptors also suggests that testosterone-derived aromatases are an underappreciated factor when considering the net impact of androgen signaling on advanced vascular disease (79, 80, 96).

## Hormone-Independent Effects of Organismal Sex: The Role of the Chromosomal Complement

Sex hormones aside, the sex chromosomes–the most fundamental and intrinsic determinant of organismal sex–is also likely to be a significant determinant of propensity for cardiovascular calcification. Both X and Y chromosomes have strong linkage associations with cardiovascular disease risk factors such as hypertension, cardiovascular inflammation, immune biology and macrophage function, and organismal metabolism (116–120). Perhaps most critically, cells derived from XX or XY organisms which are treated with identical *in vitro* conditions show differences in proliferation, fibrosis, and apoptosis in response to various agonists (121, 122). These changes are not restricted solely to vascular tissues, as osteogenic signaling and responses to various agonists also differ in aortic valve interstitial cells from XX and XY animals (123). Thus, while the Y chromosome may be referred to as a “non-recombining desert” in some biological circles (124), its sustained phenotypic impact is of undeniable importance in cardiovascular tissues.

## Conceptual Gaps and Controversies in the Field of Cardiovascular Calcification

While tremendous advances have been made in our understanding of cardiovascular calcification over the past several decades, several major gaps remain in our efforts to translate and apply both biological and clinical discoveries to the care of an individual patient.

As appropriate with the scope of this review, one could readily argue that the field's greatest gap relates to our understanding of the impact of biological sex and the sex steroid hormonal milieu on phenotypic and clinical outcomes in diseases where vascular, valvular, or microvascular calcification are of clinical importance. As the role of this critical context becomes clearer with appropriately controlled and sex-balanced pre-clinical and clinical investigation (125–127), we will undoubtedly gain deeper insights into both the pathobiological underpinnings and potential efficacious therapeutic interventions in men and women suffering from calcific cardiovascular diseases.

Perhaps the greatest controversy in the field of cardiovascular calcification—which is not necessarily exclusive from of our understanding of biological sex—is the contexts in which ectopic calcification is driven by non-osteogenic or osteogenic mechanisms. More specifically, while an overwhelming body of evidence suggests that the osteogenic signaling cascades described in this review are present in calcifying tissues from the vast majority of patients with cardiovascular calcification, clinical observations at the time of surgery or autopsy suggest that bone matrix is only evident in a relatively small fraction of this patient population (e.g., 15–25%) (128). Thus, how the cellular decision is made to initiate maladaptive, osteogenic “response to injury” at the earliest stages of microcalcific nodule formation (129) that propagates to true “ectopic bone” or alternatively expands due to progressive and persistent cellular apoptosis to form an amorphous, calcific deposit (130–133) with merely associative increases in osteogenic signaling remains remarkably elusive.

Finally, the role of the biological context of organismal age (and its fundamental biological determinants including changes in sex steroid hormones) in dictating these decisions is only beginning to be understood. While there has been a longstanding association between cellular senescence and tissue calcification [stemming from seminal work by Shanahan et al. (134–137)] more recent work suggests that the pharmacological targeting and/or clearance of senescent cells may also be a viable strategy for slowing progression of vascular calcification and dysfunction (136). The postulate that targeting fundamental biological mechanisms of aging may be a viable strategy to delay onset or prevent progression of cardiovascular calcification is supported by intriguing observation that biomarkers of biological age (e.g., telomere length) are stronger predictors of incidence of valvular heart disease than chronological age (138).

## Perspectives on the Future of the Field of Cardiovascular Calcification and Strategies for Addressing the Effects of Sex

While the field of cardiovascular calcification has made tremendous strides in advancing our understanding of osteogenic and non-osteogenic mechanisms contributing to ectopic calcium accrual over the past two decades, it is our opinion that the greatest advances—both scientifically and clinically—will be made in the near future by exploring the context of sex and sex hormones in these phenomena. While many may consider this to be too bold of a statement, it stands on a firm foundation of both clinical and biological reports demonstrating clear sex- and sex hormone-driven differences in the progression of calcific cardiovascular diseases and a glaring lack of success in viable pharmacological strategies to mitigate cardiovascular calcification in elderly persons.

While the National Institutes of Health now mandates consideration of sex as a biological variable in all studies that receive funding (e.g., from pre-clinical animal investigation to human trials), there have not been consistent requirements from publishers and journals requiring reporting of data by sex. Consequently, we would make the following additional suggestions to drive discussion of true “best practices” and accelerate development of critical insights into mechanisms underlying cardiovascular calcification in future studies. First, we suggest that *in vitro* studies should include independent cell lines derived from each sex of the species being studied, which will allow for characterization of the impact of chromosomal complement and differing epigenetic demarcations in the absence of the sex-steroid and systemic hormonal millieu. With the emergence of evidence that cell line immortalization can drive X and Y chromosomal reconfiguration (139) and the absence of data demonstrating whether such phenomena eliminates sex-dependent molecular responses to exogenous stressors, we also advocate for the use of primary cell lines until comprehensive characterizations of cell phenotype and response are available. Second, we suggest that all sex-disaggregated data should be available within manuscripts and/or online supplements, and that studies should be designed to detect sex differences with appropriate statistical power in an a priori manner. In clinical conditions in which a disease occurs predominantly in one sex vs. the other, we feel a minimum recommendation of having the experimental sample composition be reflective of the sex distribution within the patient population of interest is reasonable. Finally, execution of appropriate hormonal depletion, repletion, or crossover studies would represent a major advance in the field. By appropriate, we refer not only to absolute hormonal levels but also to timing of repletion/depletion (i.e., initiation of the insult later in life, similar to what occurs in humans).

While we believe few investigators would argue that the abovementioned recommendations directly align with the foundational principles of scientific rigor, we are acutely aware that the logistics of implementing such recommendations must be addressed. While doubling the scope and scale of ongoing research projects is neither feasible nor sustainable, we would argue that generation of pre-clinical and clinical datasets that do not inform either the pathobiological underpinnings or clinical care of half of the world's population reduces the relevance and impact of scientific investigation around the globe. At present, we believe there are few viable arguments against the intentional and appropriate inclusion (and subsequent disaggregated presentation) of data from both sexes in studies of cardiovascular calcification, as the number of samples required for demonstrating sex differences should not increase dramatically when truly qualitatively different trends are uncovered. We acknowledge that discovery of such dichotomous sex responses can often spur new lines of investigation that are beyond the scope of the existing project and subsequently require support through additional funding mechanisms. Critically, we commend NIH and several other funding entities for creation of supplemental research awards devoted to supporting more detailed investigation of mechanisms underlying unexpectedly discovered sex differences, as well as creation of recent RFA's that prioritize identification of novel sex differences in cardiovascular calcification and disease.

Ultimately, we feel that appropriately designed and executed clinical studies—including both men and women potentially with independent outcome measures in each sex (e.g., not only valvular function as a primary outcome but predominantly calcification in men and fibrosis in women as secondary outcomes) is an essential step in ensuring that the utility of sex in predicting therapeutic efficacy and effectiveness across the translational spectrum. While beyond the scope of this review [and covered recently in Sritharen et al. (14)], emerging data strongly suggest that incoming risk profiles, outcomes for surgical valve replacement, outcomes for transcatheter valve replacement, and comorbid condition frequency following disease diagnosis differ robustly amongst men and women, and can serve as a critical catalyst for driving impactful investigation in the biological mechanisms underlying such observations.

## Summary

The clinical presentation, biological underpinnings, and molecular interactions with sex hormones and biological sex, and ultimate strategies to therapeutically prevent or slow progression of cardiovascular calcification differ dramatically between men and women. While in the United States, the National Institutes of Health (NIH) mandate for inclusion of both sexes in research will undoubtedly serve to advance our understanding of these differences, it is our hope that this review will spur additional genuine interest in understanding critical biological and clinical contexts—including, but not limited to organismal sex—and drive transformative advances in the strategies and tools needed to reduce the growing global burden of calcific vascular and valvular diseases.

## Author Contributions

All authors contributed to the drafting and revision of the manuscript and concepts expressed herein.

### Conflict of Interest Statement

The authors declare that the research was conducted in the absence of any commercial or financial relationships that could be construed as a potential conflict of interest.
